# Case Report of COVID-19 Positive Male with Late-Onset Full Body Maculopapular Rash

**DOI:** 10.21980/J86W72

**Published:** 2021-01-15

**Authors:** Sarah Harirforoosh, Jessica Hoffmann, Emily Bernal

**Affiliations:** * University of California, Irvine, Department of Medicine, Orange, CA; ^ University of California, Irvine, Department of Emergency Medicine, Orange, CA; † University of California, Irvine, Irvine, CA

## Abstract

Each day, novel information is being learned about Coronavirus disease 19 (COVID-19), a disease increasingly seen in the emergency department (ED). Our case adds to reports of a maculopapular rash among the newer symptoms to be aware of in diagnosing COVID-19. A 35-year-old male presented with an 11-day history of fever, dry cough, diarrhea, and change in taste. He endorsed two known exposures to COVID-19 and had previously tested negative twice for the virus. The patient additionally complained of a whole-body, pruritic rash that presented six days after his initial symptoms. A workup was completed for the patient’s fever and labs only revealed a potassium of 3.3 mEq/L. A chest X-ray showed right basilar patchy opacities consistent with pneumonia, and the patient tested positive for COVID-19 and negative for Epstein-Barr Virus, a commonly missed culprit in those with a rash, via polymerase chain reaction (PCR). The patient’s vitals were stable, and he was discharged with supportive measures at home. COVID-19 was determined as a likely etiology for the rash. With new insights into COVID-19 every day, we demonstrate a case of viral exanthema as a potential presenting manifestation in a COVID-19 patient and one that medical providers should be familiar with in these patients in the ED.

**Topics:**

COVID-19, dermatology, infectious disease, viral exanthema.

**Figure:**
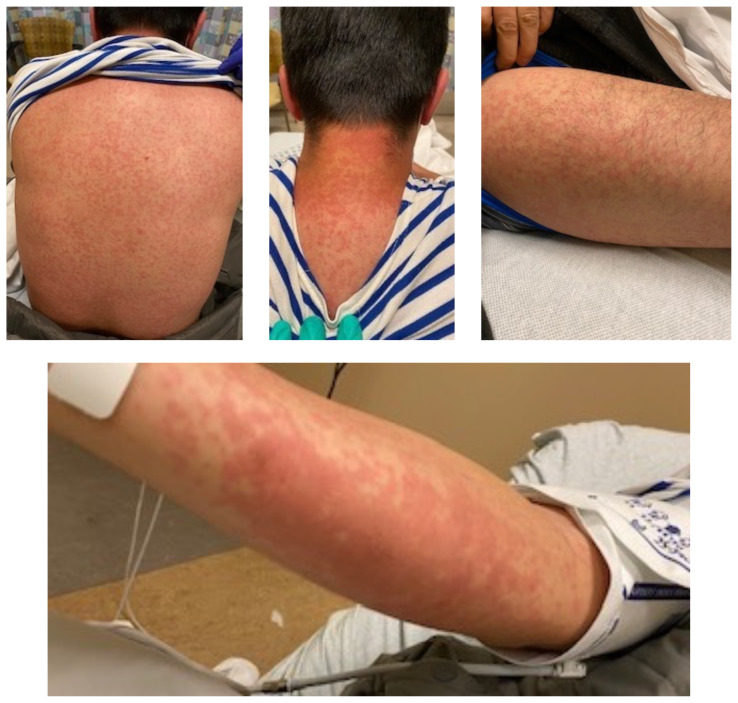

## Brief introduction

A variety of skin conditions, including rash and other nonspecific skin eruptions, account for 3.3% of visits to the emergency department (ED).^1^ These rashes can vary in their underlying etiology, but some can point towards serious diagnoses. COVID-19 (illness caused by SARS-CoV-2) was declared a pandemic by the World Health Organization (WHO) in March 2020, and the number of cases continues to increase on a global scale.^2^ In addition to the respiratory symptoms that have been well documented, only recently have reports emerged on the varying cutaneous manifestations of COVID-19. To date, the skin lesions in COVID-19 have most commonly been described as maculopapular eruptions, acral erythema with vesicles or pustules, urticarial lesions, other vesicular exanthema, or livedo pattern that have lasted minutes to weeks.^3^ The rashes have been most frequently identified on the trunk and hands and feet with several cases improving or resolving spontaneously.^4^,^5^ This unique case of widespread rash highlights the importance of considering a presenting exanthema as a component in the diagnosis of COVID-19 in the ED.

## Presenting concerns and clinical findings

This is a case of a 35-year-old male with a history of hyperlipidemia who presented to the ED with fever and rash. He reported 11 days of symptoms, beginning with headache and fever (maximum temperature 102 degrees Fahrenheit). Additionally, the patient endorsed dry cough, diarrhea, body aches, and change in sensation of taste. Four days prior to presenting to the ED, the patient began having a rash in his extremities, which progressed to his torso and face. The rash was characterized as pruritic and painless. He reported two known COVID-19 exposures, including his roommate and coworker. The patient spent two days with his co-worker, who at the time was symptomatic, and reported constant contact with his roommate. He previously tested negative for COVID-19 twice while symptomatic. The first test was on day one of symptoms, and the second was on day seven of symptoms. The patient denied change in diet, recent travel, or allergies, and was up to date on his vaccinations. Of note, on day one of symptoms, the patient was prescribed a 10-day course of amoxicillin for tonsillar exudates. He reported prior use of amoxicillin without drug reaction.

## Significant findings

The images demonstrate a diffuse, flat, maculopapular exanthema along the torso, bilateral upper and lower extremities, and neck without edema consistent with reported cutaneous manifestations of COVID-19. There are no surrounding bullae, vesicles, or draining. On palpation, there was blanching of the rash. Sensation to light touch was intact in all extremities. The findings were also apparent on the face with no mucosal involvement.

## Patient course

A workup for the patient’s symptoms was obtained while in the ED. Two negative COVID-19 PCR tests were charted prior to the patient’s current presentation. However, COVID-19 was detected at this visit. All three tests were completed using cobas 6800 SARS-CoV-2 quantitative assay from Roche. A potassium of 3.3 mEq/L was the only abnormal laboratory value. Lactate and blood cultures were obtained to rule out a critically acute process and were normal (1.1 mEq/L) and negative for growth, respectively. Chest X-ray showed new right basilar patchy opacities concerning for pneumonia secondary to COVID-19 or community acquired pneumonia (CAP). As there was concern for the patient’s rash being secondary to Epstein-Barr Virus (EBV) mononucleosis, an EBV PCR was obtained, which was negative. Vital signs were within normal limits; the patient maintained an oxygen saturation greater than 95% on room air and was not dyspneic. Amoxicillin was held due to consideration of a potential drug reaction. He was discharged on azithromycin for CAP and counseled on continuing supportive measures at home.

## Discussion

This case showcases the differential included in the presentation of maculopapular rash and its relation to COVID-19, a disease that continues to significantly impact the medical field. As research evolves around COVID-19, a case such as this adds to reports of the various rashes to be aware of in the ED when evaluating for this disease. The morbilliform rash demonstrated diffusely throughout our patient may be due to a wide etiology. A cutaneous drug eruption is included on that differential. Specifically, exanthematous drug reactions are a well-documented adverse effect to penicillins, cephalosporins, sulfonamides, antimicrobials, allopurinol, and numerous anticonvulsants.^6^ These rashes are often mild and difficult to distinguish from viral exanthemas. Moreover, among the category of viral exanthemas that cause a maculopapular rash, depending on the virus, mucous membranes may or may not be involved. Primary Herpes Simplex Virus-1 and 2, acute EBV, Rubella, influenza, Barmah Forest virus, and HHV-8 can present as a maculopapular rash in various distributions along the body.^7^ Nonetheless, COVID-19 should now be included on that list.

Among the cutaneous manifestations of COVID-19, morbilliform rash is the most common morphology identified at this point.^8^ The rash may also present in the form of urticaria, vesicular eruptions, acral lesions, livedoid eruptions, or as a combination of these. To date, postulated underlying mechanisms of the cutaneous manifestations include direct viral binding or secondarily through allergic-immunologic mediated processes.^9^ Moreover, several early papers stress the fact that it is difficult to pinpoint whether the rash is due to COVID-19 itself or as a reaction to a medication since many times patients will have overlap of the two.^5^ Additionally, preliminary literature review characterizes the rash as spontaneously resolving in 10 days; however, in several cases, oral antihistamines and local corticosteroids have been utilized as a treatment.^4^

To summarize, this case features a presenting symptom of rash in a patient with characteristic COVID-19 symptoms. Maculopapular rash is among the most common form of cutaneous reaction in this disease, as we exemplify in our patient. In general, an exanthema of this nature can be due to many etiologies, including drug eruptions and additional viral exanthemas. For our patient, due to distinctive symptoms for COVID-19, endorsing no prior reaction to amoxicillin usage, being up to date on his vaccinations, and having a negative EBV screen, we concluded that his widespread maculopapular exanthema was most likely secondary to confirmed COVID-19. However, as noted, a drug reaction to amoxicillin and viral exanthema secondary to early EBV infection were included as differential diagnoses. The primary take-away of this case is the recognition of diffuse rash as a component in the diagnosis of COVID-19.

## Supplementary Information








