# Supplementary figures and images for: Case Report of COVID-19 Positive Male with Late-Onset Full Body Maculopapular Rash
Source: J Educ Teach Emerg Med. 2021 Jan 15;6(1):V19–22. doi: 10.21980/J86W72 (PMC10332756; doi:10.21980/J86W72)

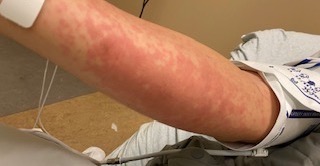

Supplement: Supplementary file 1 [file jetem-6-1-v19-supp1.jpg]

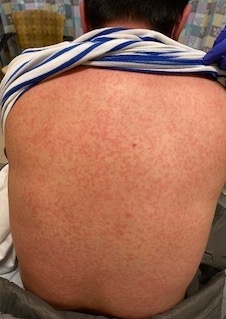

Supplement: Supplementary file 2 [file jetem-6-1-v19-supp2.jpg]

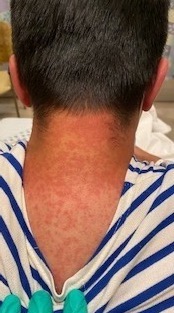

Supplement: Supplementary file 3 [file jetem-6-1-v19-supp3.jpg]

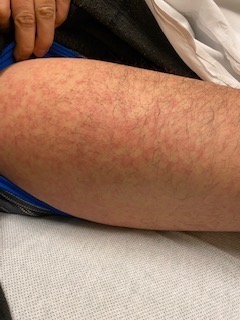

Supplement: Supplementary file 4 [file jetem-6-1-v19-supp4.jpg]
